# Elucidating calpain’s role in the breast cancer cell signalling network

**DOI:** 10.1186/1753-6561-7-S2-P66

**Published:** 2013-04-04

**Authors:** Stacy Visser-Grieve, Jing Hu, Peter Greer

**Affiliations:** 1 Department of Pathology and Molecular Medicine, Queen’s University, Kingston, ON, Canada; 2 Division of Cancer Biology and Genetics, Queen’s Cancer Research Institute, Kingston, ON, Canada

## Background

Improved outcomes for breast cancer patients include the use of novel targeted agents acting on key survival and proliferation pathways. Calpain is an intracellular calcium regulated protease with two predominant isoforms, calpain 1 and calpain 2. The cleavage of target proteins by calpain 1 or 2 regulates their cellular functions, including proliferation, survival and migration. We hypothesize that different survival outcomes in cancer cells in response to various therapeutics relate to distinct profiles of signaling proteins being cleaved by calpain. Thus, inhibition of calpain could interact additively or synergistically with specific therapeutics to improve breast cancer outcomes. To realize this possibility, it is essential to identify profiles of calpain targets cleaved upon challenge with specific therapeutics and determine the functional effects of calpain-mediated cleavage.

## Materials and methods

Overexpression or knock-down of calpain 1 and 2 was accomplished in human breast cancer cell lines including MDA-MB-231, SKBR3, and BT474 cells through the use of lentiviral expression systems. Assessment of the functional implication of modulating calpain expression included both proliferation and apoptotic assays. Future work utilizing large-scale proteome screens as well as preclinical cell and animal models will identify calpain targets and global cellular signaling changes when calpain expression and activity are altered.

## Results

In human breast cancer cell lines, overexpression of calpain, but not an inactive mutant leads to increased cell proliferation and renders breast cancer cells expressing the HER2 oncogene resistant to a HER2 specific drug, Trastuzumab. In addition, loss of calpain expression leads to reduced cell proliferation and decreased ability to grow tumors in mice.

## Conclusions

Results from this study provide evidence that calpain functions as an oncogene and plays a role in regulating cell survival in response to Trastuzumab. This study can lead to the discovery of drug combinations for the improvement of breast cancer treatment.

## Financial support

This work was supported by the Canadian Institutes of Health Research to P.G., the Queen’s University Terry Fox Foundation Training Program in Transdisciplinary Cancer Research in partnership with CIHR and the Canadian Breast Cancer Foundation to S.VG.

